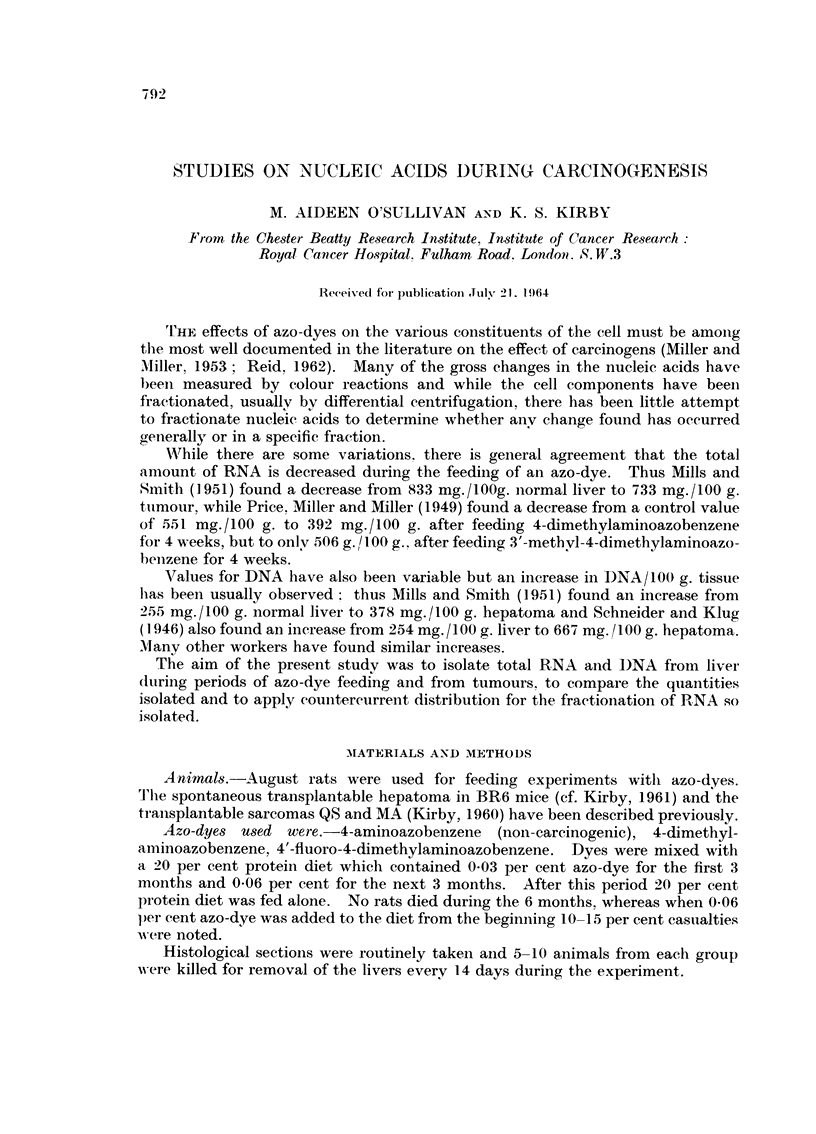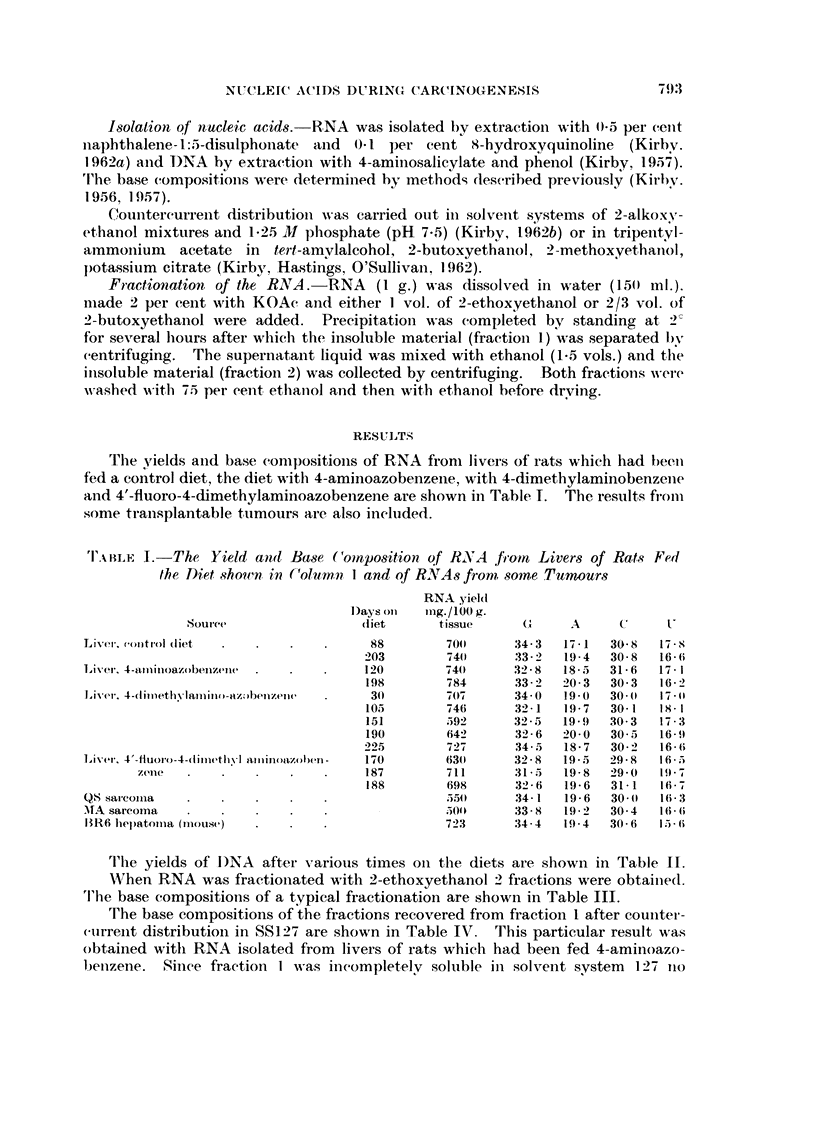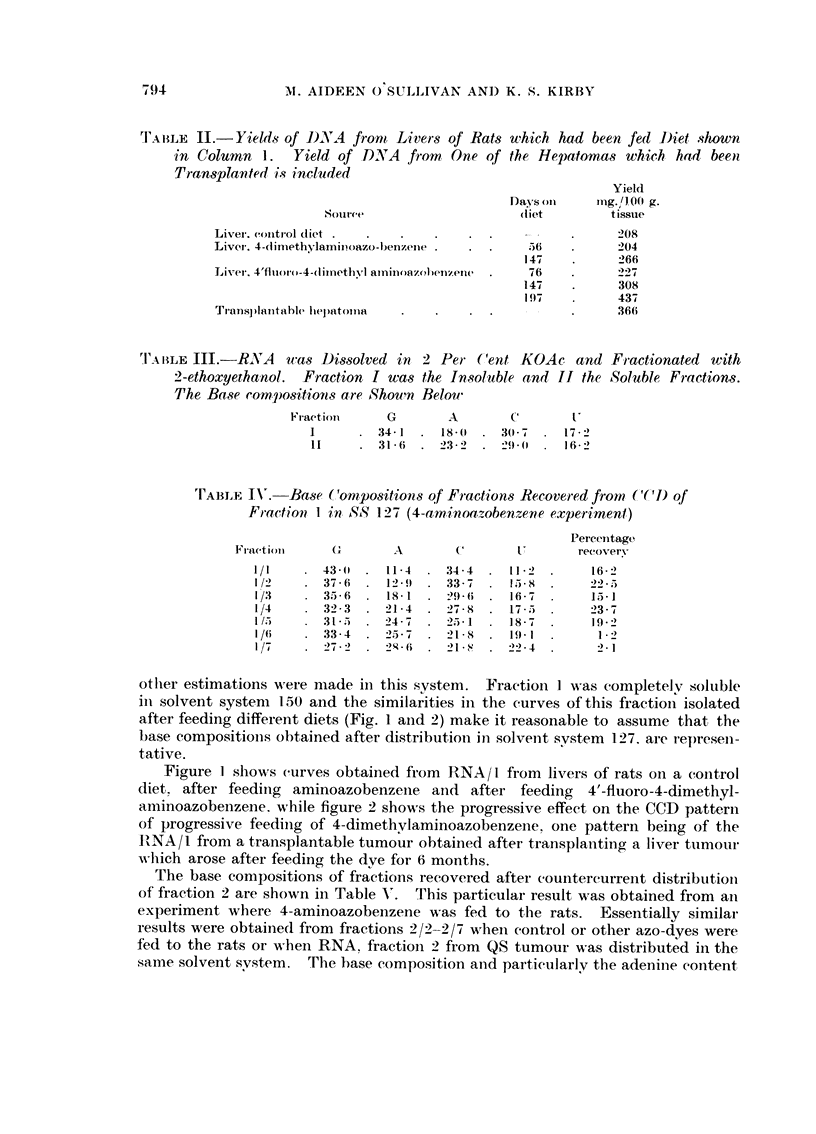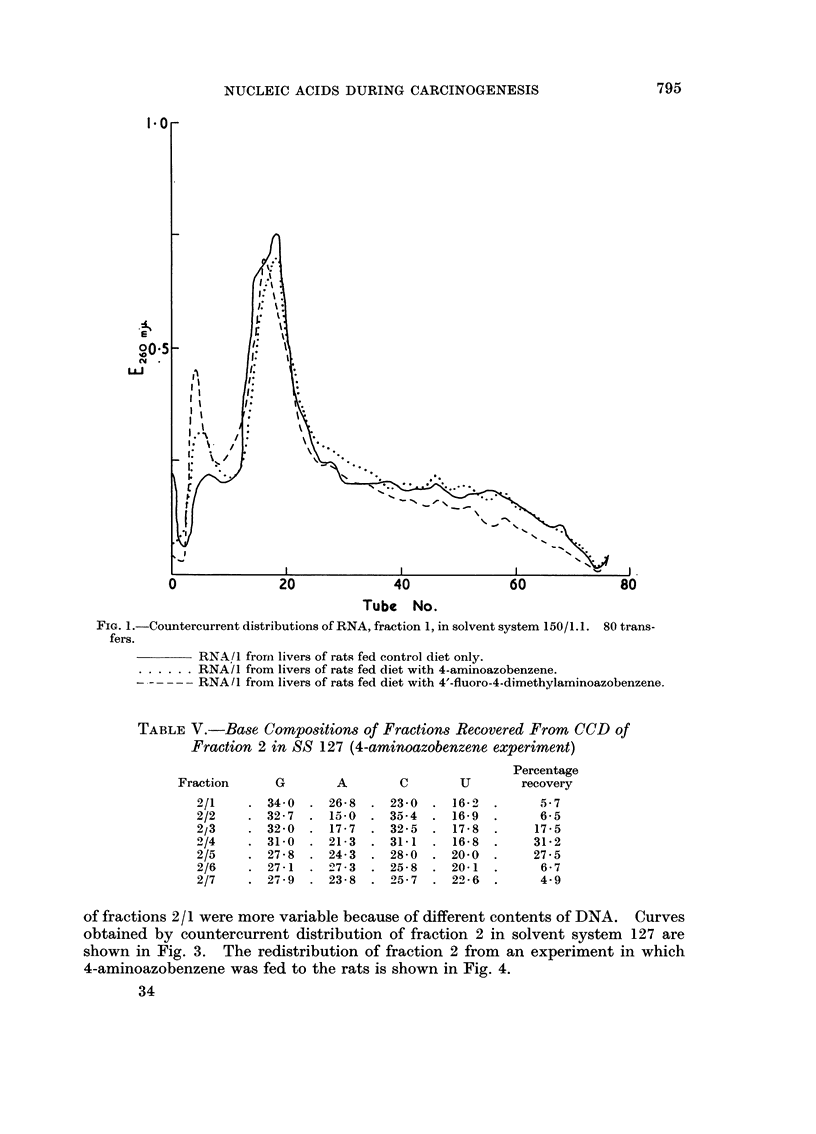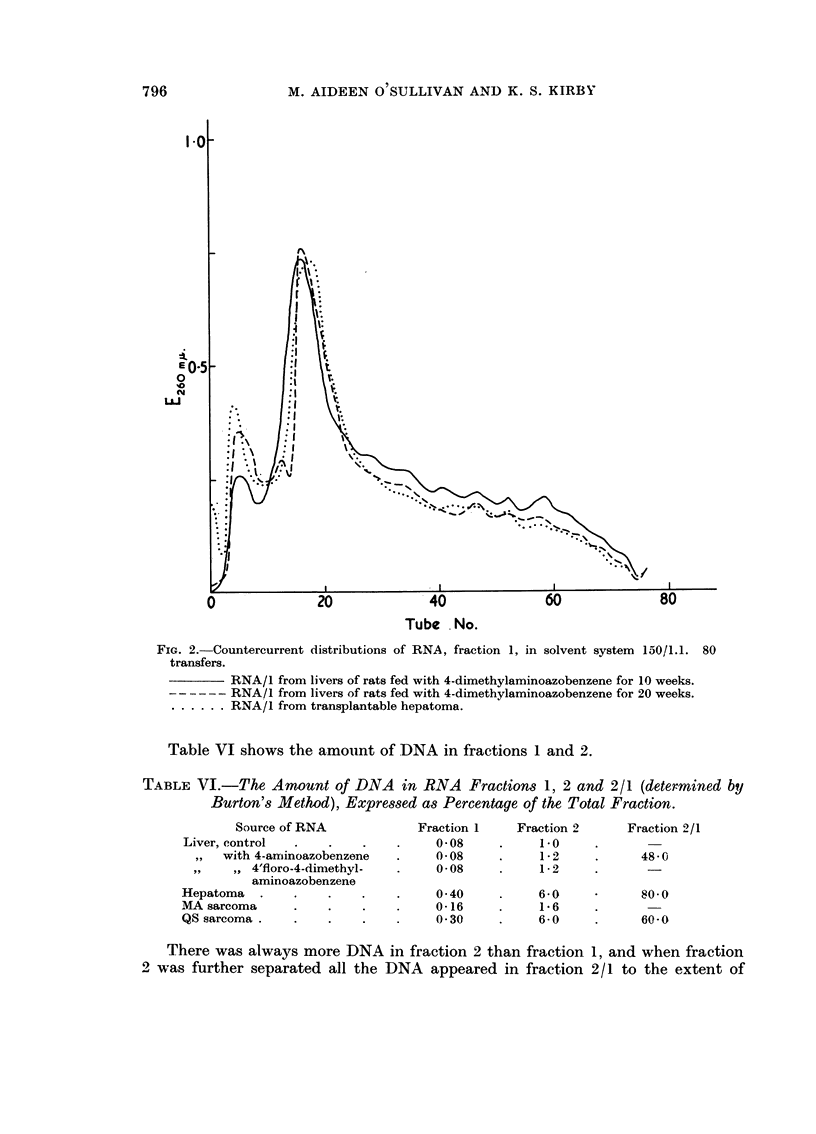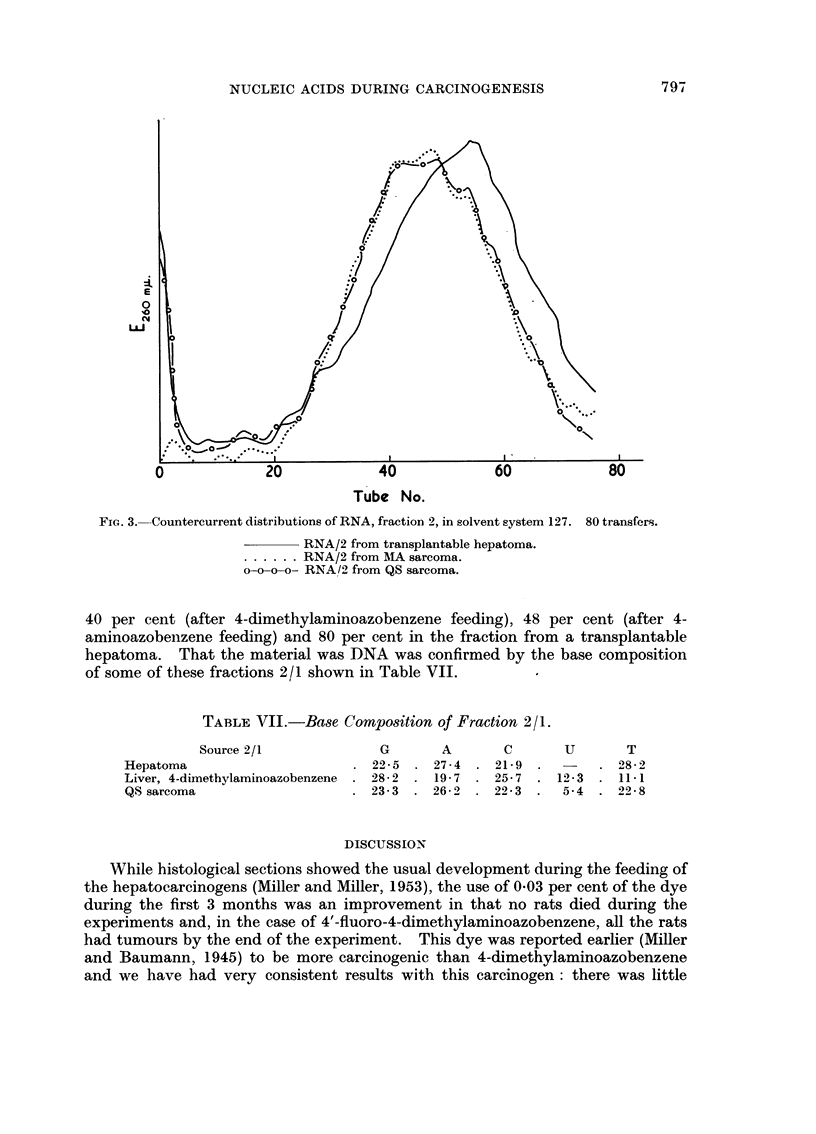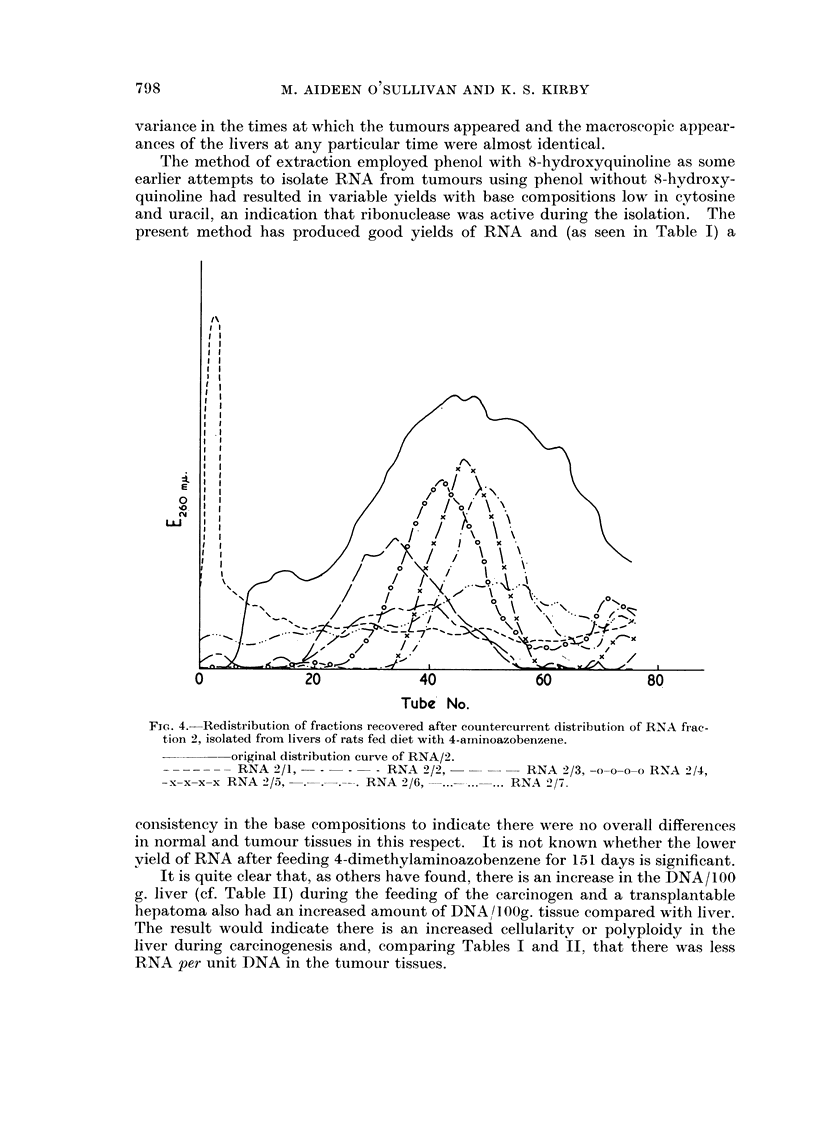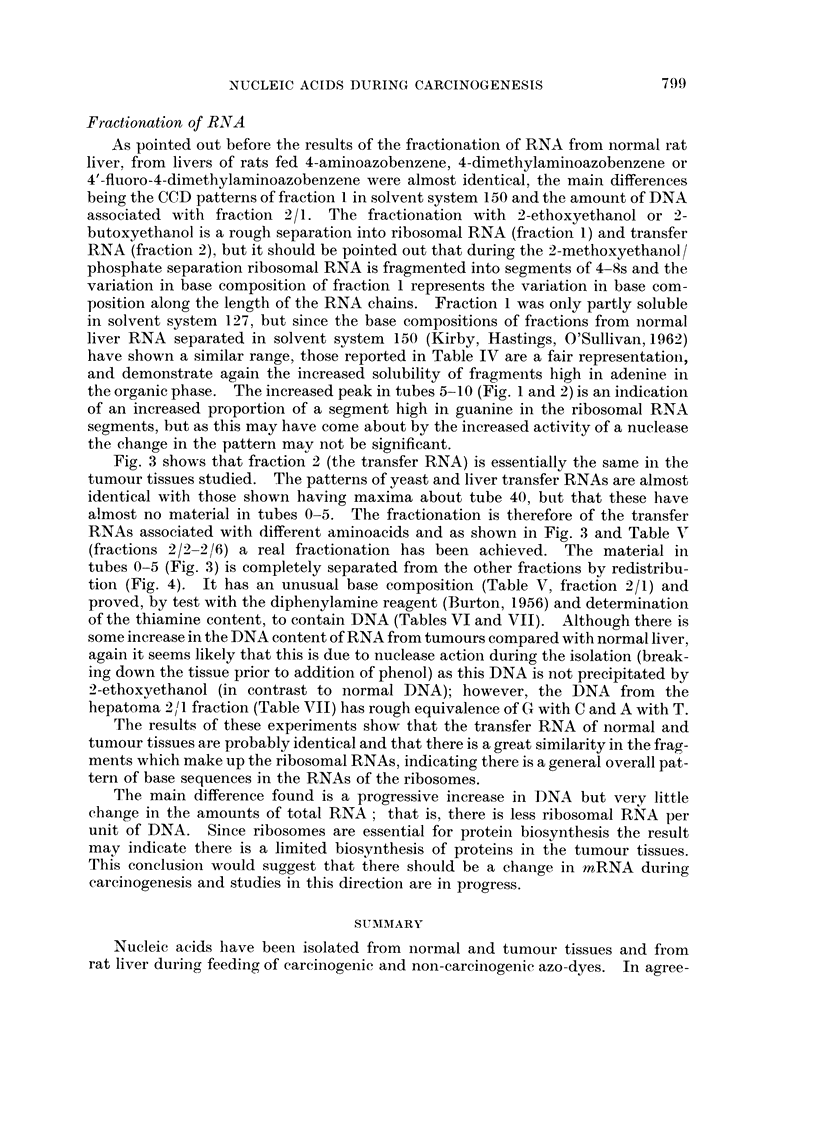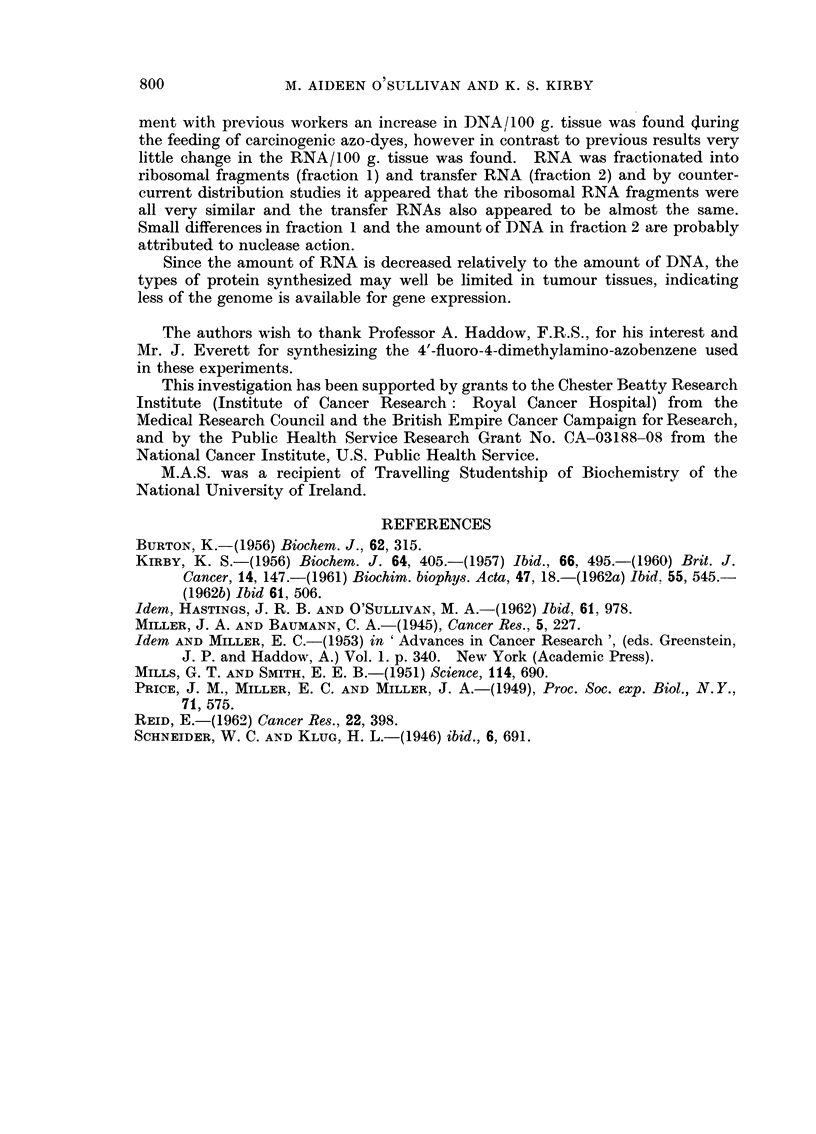# Studies on Nucleic Acids During Carcinogenesis

**DOI:** 10.1038/bjc.1964.92

**Published:** 1964-12

**Authors:** M. Aideen O'Sullivan, K. S. Kirby


### STUDIES ON NUCLEIC ACIDS DURING CARCINOGENESIS

M. AIDEEN O'SULLIVAN AND K. S. KIRBY

*From the Chester Beatty Research Institute, Institute of Cancer Research: Royal Cancer Hospital, Fulham Road, London, S.W.3*

Received for publication July 21, 1964

THE effects of azo-dyes on the various constituents of the cell must be among the most well documented in the literature on the effect of carcinogens (Miller and Miller, 1953; Reid, 1962). Many of the gross changes in the nucleic acids have been measured by colour reactions and while the cell components have been fractionated, usually by differential centrifugation, there has been little attempt to fractionate nucleic acids to determine whether any change found has occurred generally or in a specific fraction.

While there are some variations, there is general agreement that the total amount of RNA is decreased during the feeding of an azo-dye. Thus Mills and Smith (1951) found a decrease from 833 mg./100g. normal liver to 733 mg./100 g. tumour, while Price, Miller and Miller (1949) found a decrease from a control value of 551 mg./100 g. to 392 mg./100 g. after feeding 4-dimethylaminoazobenzene for 4 weeks, but to only 506 g./100 g., after feeding 3′-methyl-4-dimethylaminoazobenzene for 4 weeks.

Values for DNA have also been variable but an increase in DNA/100 g. tissue has been usually observed: thus Mills and Smith (1951) found an increase from 255 mg./100 g. normal liver to 378 mg./100 g. hepatoma and Schneider and Klug (1946) also found an increase from 254 mg./100 g. liver to 667 mg./100 g. hepatoma. Many other workers have found similar increases.

The aim of the present study was to isolate total RNA and DNA from liver during periods of azo-dye feeding and from tumours, to compare the quantities isolated and to apply countercurrent distribution for the fractionation of RNA so isolated.

### MATERIALS AND METHODS

*Animals.*—August rats were used for feeding experiments with azo-dyes. The spontaneous transplantable hepatoma in BR6 mice (cf. Kirby, 1961) and the transplantable sarcomas QS and MA (Kirby, 1960) have been described previously.

*Azo-dyes used were.*—4-aminoazobenzene (non-carcinogenic), 4-dimethylaminoazobenzene, 4′-fluoro-4-dimethylaminoazobenzene. Dyes were mixed with a 20 per cent protein diet which contained 0·03 per cent azo-dye for the first 3 months and 0·06 per cent for the next 3 months. After this period 20 per cent protein diet was fed alone. No rats died during the 6 months, whereas when 0·06 per cent azo-dye was added to the diet from the beginning 10–15 per cent casualties were noted.

Histological sections were routinely taken and 5–10 animals from each group were killed for removal of the livers every 14 days during the experiment.

*Isolation of nucleic acids.*—RNA was isolated by extraction with 0·5 per cent naphthalene-1:5-disulphonate and 0·1 per cent 8-hydroxyquinoline (Kirby, 1962*a*) and DNA by extraction with 4-aminosalicylate and phenol (Kirby, 1957). The base compositions were determined by methods described previously (Kirby, 1956, 1957).

Countercurrent distribution was carried out in solvent systems of 2-alkoxy-ethanol mixtures and 1·25 *M* phosphate (pH 7·5) (Kirby, 1962*b*) or in tripentyl-ammonium acetate in *tert*-amylalcohol, 2-butoxyethanol, 2-methoxyethanol, potassium citrate (Kirby, Hastings, O'Sullivan, 1962).

*Fractionation of the RNA.*—RNA (1 g.) was dissolved in water (150 ml.), made 2 per cent with KOAc and either 1 vol. of 2-ethoxyethanol or 2/3 vol. of 2-butoxyethanol were added. Precipitation was completed by standing at 2° for several hours after which the insoluble material (fraction 1) was separated by centrifuging. The supernatant liquid was mixed with ethanol (1·5 vols.) and the insoluble material (fraction 2) was collected by centrifuging. Both fractions were washed with 75 per cent ethanol and then with ethanol before drying.

### RESULTS

The yields and base compositions of RNA from livers of rats which had been fed a control diet, the diet with 4-aminoazobenzene, with 4-dimethylaminobenzene and 4′-fluoro-4-dimethylaminoazobenzene are shown in Table I. The results from some transplantable tumours are also included.

TABLE I.—*The Yield and Base Composition of RNA from Livers of Rats Fed the Diet shown in Column 1 and of RNAs from some Tumours*

| Source | Days on diet | RNA yield mg./100 g. tissue | G | A | C | U |
|---|---|---|---|---|---|---|
| Liver, control diet . . . . | 88 | 700 | 34·3 | 17·1 | 30·8 | 17·8 |
| | 203 | 740 | 33·2 | 19·4 | 30·8 | 16·6 |
| Liver, 4-aminoazobenzene . . . | 120 | 740 | 32·8 | 18·5 | 31·6 | 17·1 |
| | 198 | 784 | 33·2 | 20·3 | 30·3 | 16·2 |
| Liver, 4-dimethylamino-azobenzene . | 30 | 707 | 34·0 | 19·0 | 30·0 | 17·0 |
| | 105 | 746 | 32·1 | 19·7 | 30·1 | 18·1 |
| | 151 | 592 | 32·5 | 19·9 | 30·3 | 17·3 |
| | 190 | 642 | 32·6 | 20·0 | 30·5 | 16·9 |
| | 225 | 727 | 34·5 | 18·7 | 30·2 | 16·6 |
| Liver, 4′-fluoro-4-dimethyl aminoazoben-zene . . . . . . | 170 | 630 | 32·8 | 19·5 | 29·8 | 16·5 |
| | 187 | 711 | 31·5 | 19·8 | 29·0 | 19·7 |
| | 188 | 698 | 32·6 | 19·6 | 31·1 | 16·7 |
| QS sarcoma . . . . . . | | 550 | 34·1 | 19·6 | 30·0 | 16·3 |
| MA sarcoma . . . . . . | | 500 | 33·8 | 19·2 | 30·4 | 16·6 |
| BR6 hepatoma (mouse) . . . | | 723 | 34·4 | 19·4 | 30·6 | 15·6 |

The yields of DNA after various times on the diets are shown in Table II.

When RNA was fractionated with 2-ethoxyethanol 2 fractions were obtained. The base compositions of a typical fractionation are shown in Table III.

The base compositions of the fractions recovered from fraction 1 after counter-current distribution in SS127 are shown in Table IV. This particular result was obtained with RNA isolated from livers of rats which had been fed 4-aminoazo-benzene. Since fraction 1 was incompletely soluble in solvent system 127 no

TABLE II.—*Yields of DNA from Livers of Rats which had been fed Diet shown in Column 1. Yield of DNA from One of the Hepatomas which had been Transplanted is included*

| Source | Days on diet | Yield mg./100 g. tissue |
|---|---|---|
| Liver, control diet | — | 208 |
| Liver, 4-dimethylaminoazo-benzene | 56 | 204 |
| | 147 | 266 |
| Liver, 4'fluoro-4-dimethyl aminoazobenzene | 76 | 227 |
| | 147 | 308 |
| | 197 | 437 |
| Transplantable hepatoma | — | 366 |

TABLE III.—*RNA was Dissolved in 2 Per Cent KOAc and Fractionated with 2-ethoxyethanol. Fraction I was the Insoluble and II the Soluble Fractions. The Base compositions are Shown Below*

| Fraction | G | A | C | U |
|---|---|---|---|---|
| I | 34·1 | 18·0 | 30·7 | 17·2 |
| II | 31·6 | 23·2 | 29·0 | 16·2 |

TABLE IV.—*Base Compositions of Fractions Recovered from CCD of Fraction 1 in SS 127 (4-aminoazobenzene experiment)*

| Fraction | G | A | C | U | Percentage recovery |
|---|---|---|---|---|---|
| 1/1 | 43·0 | 11·4 | 34·4 | 11·2 | 16·2 |
| 1/2 | 37·6 | 12·9 | 33·7 | 15·8 | 22·5 |
| 1/3 | 35·6 | 18·1 | 29·6 | 16·7 | 15·1 |
| 1/4 | 32·3 | 21·4 | 27·8 | 17·5 | 23·7 |
| 1/5 | 31·5 | 24·7 | 25·1 | 18·7 | 19·2 |
| 1/6 | 33·4 | 25·7 | 21·8 | 19·1 | 1·2 |
| 1/7 | 27·2 | 28·6 | 21·8 | 22·4 | 2·1 |

other estimations were made in this system. Fraction 1 was completely soluble in solvent system 150 and the similarities in the curves of this fraction isolated after feeding different diets (Fig. 1 and 2) make it reasonable to assume that the base compositions obtained after distribution in solvent system 127, are representative.

Figure 1 shows curves obtained from RNA/1 from livers of rats on a control diet, after feeding aminoazobenzene and after feeding 4'-fluoro-4-dimethylaminoazobenzene, while figure 2 shows the progressive effect on the CCD pattern of progressive feeding of 4-dimethylaminoazobenzene, one pattern being of the RNA/1 from a transplantable tumour obtained after transplanting a liver tumour which arose after feeding the dye for 6 months.

The base compositions of fractions recovered after countercurrent distribution of fraction 2 are shown in Table V. This particular result was obtained from an experiment where 4-aminoazobenzene was fed to the rats. Essentially similar results were obtained from fractions 2/2–2/7 when control or other azo-dyes were fed to the rats or when RNA, fraction 2 from QS tumour was distributed in the same solvent system. The base composition and particularly the adenine content

FIG. 1.—Countercurrent distributions of RNA, fraction 1, in solvent system 150/1.1. 80 transfers.

——— RNA/1 from livers of rats fed control diet only.
...... RNA/1 from livers of rats fed diet with 4-aminoazobenzene.
– – – – RNA/1 from livers of rats fed diet with 4′-fluoro-4-dimethylaminoazobenzene.

TABLE V.—*Base Compositions of Fractions Recovered From CCD of Fraction 2 in SS 127 (4-aminoazobenzene experiment)*

| Fraction | G | A | C | U | Percentage recovery |
|---|---|---|---|---|---|
| 2/1 | 34·0 | 26·8 | 23·0 | 16·2 | 5·7 |
| 2/2 | 32·7 | 15·0 | 35·4 | 16·9 | 6·5 |
| 2/3 | 32·0 | 17·7 | 32·5 | 17·8 | 17·5 |
| 2/4 | 31·0 | 21·3 | 31·1 | 16·8 | 31·2 |
| 2/5 | 27·8 | 24·3 | 28·0 | 20·0 | 27·5 |
| 2/6 | 27·1 | 27·3 | 25·8 | 20·1 | 6·7 |
| 2/7 | 27·9 | 23·8 | 25·7 | 22·6 | 4·9 |

of fractions 2/1 were more variable because of different contents of DNA. Curves obtained by countercurrent distribution of fraction 2 in solvent system 127 are shown in Fig. 3. The redistribution of fraction 2 from an experiment in which 4-aminoazobenzene was fed to the rats is shown in Fig. 4.

FIG. 2.—Countercurrent distributions of RNA, fraction 1, in solvent system 150/1.1. 80 transfers.

———— RNA/1 from livers of rats fed with 4-dimethylaminoazobenzene for 10 weeks.
– – – – – – RNA/1 from livers of rats fed with 4-dimethylaminoazobenzene for 20 weeks.
. . . . . . RNA/1 from transplantable hepatoma.

Table VI shows the amount of DNA in fractions 1 and 2.

TABLE VI.—*The Amount of DNA in RNA Fractions* 1, 2 *and* 2/1 (*determined by Burton's Method*), *Expressed as Percentage of the Total Fraction.*

| Source of RNA | Fraction 1 | Fraction 2 | Fraction 2/1 |
|---|---|---|---|
| Liver, control | 0·08 | 1·0 | — |
| ,, with 4-aminoazobenzene | 0·08 | 1·2 | 48·0 |
| ,, ,, 4'floro-4-dimethyl-aminoazobenzene | 0·08 | 1·2 | — |
| Hepatoma | 0·40 | 6·0 | 80·0 |
| MA sarcoma | 0·16 | 1·6 | — |
| QS sarcoma | 0·30 | 6·0 | 60·0 |

There was always more DNA in fraction 2 than fraction 1, and when fraction 2 was further separated all the DNA appeared in fraction 2/1 to the extent of

FIG. 3.—Countercurrent distributions of RNA, fraction 2, in solvent system 127. 80 transfers.

——— RNA/2 from transplantable hepatoma.

. . . . . . RNA/2 from MA sarcoma.

o–o–o–o– RNA/2 from QS sarcoma.

40 per cent (after 4-dimethylaminoazobenzene feeding), 48 per cent (after 4-aminoazobenzene feeding) and 80 per cent in the fraction from a transplantable hepatoma. That the material was DNA was confirmed by the base composition of some of these fractions 2/1 shown in Table VII.

TABLE VII.—*Base Composition of Fraction* 2/1.

| Source 2/1 | G | A | C | U | T |
|---|---|---|---|---|---|
| Hepatoma | 22·5 | 27·4 | 21·9 | — | 28·2 |
| Liver, 4-dimethylaminoazobenzene | 28·2 | 19·7 | 25·7 | 12·3 | 11·1 |
| QS sarcoma | 23·3 | 26·2 | 22·3 | 5·4 | 22·8 |

### DISCUSSION

While histological sections showed the usual development during the feeding of the hepatocarcinogens (Miller and Miller, 1953), the use of 0·03 per cent of the dye during the first 3 months was an improvement in that no rats died during the experiments and, in the case of 4′-fluoro-4-dimethylaminoazobenzene, all the rats had tumours by the end of the experiment. This dye was reported earlier (Miller and Baumann, 1945) to be more carcinogenic than 4-dimethylaminoazobenzene and we have had very consistent results with this carcinogen : there was little variance in the times at which the tumours appeared and the macroscopic appearances of the livers at any particular time were almost identical.

The method of extraction employed phenol with 8-hydroxyquinoline as some earlier attempts to isolate RNA from tumours using phenol without 8-hydroxyquinoline had resulted in variable yields with base compositions low in cytosine and uracil, an indication that ribonuclease was active during the isolation. The present method has produced good yields of RNA and (as seen in Table I) a

FIG. 4.—Redistribution of fractions recovered after countercurrent distribution of RNA fraction 2, isolated from livers of rats fed diet with 4-aminoazobenzene.

————original distribution curve of RNA/2.
------- RNA 2/1, — · — · — · RNA 2/2, — — — — RNA 2/3, -o-o-o-o RNA 2/4, -x-x-x-x RNA 2/5, —.—.—.—. RNA 2/6, —...—...—... RNA 2/7.

consistency in the base compositions to indicate there were no overall differences in normal and tumour tissues in this respect. It is not known whether the lower yield of RNA after feeding 4-dimethylaminoazobenzene for 151 days is significant.

It is quite clear that, as others have found, there is an increase in the DNA/100 g. liver (cf. Table II) during the feeding of the carcinogen and a transplantable hepatoma also had an increased amount of DNA/100g. tissue compared with liver. The result would indicate there is an increased cellularity or polyploidy in the liver during carcinogenesis and, comparing Tables I and II, that there was less RNA *per* unit DNA in the tumour tissues.

*Fractionation of RNA*

As pointed out before the results of the fractionation of RNA from normal rat liver, from livers of rats fed 4-aminoazobenzene, 4-dimethylaminoazobenzene or 4′-fluoro-4-dimethylaminoazobenzene were almost identical, the main differences being the CCD patterns of fraction 1 in solvent system 150 and the amount of DNA associated with fraction 2/1. The fractionation with 2-ethoxyethanol or 2-butoxyethanol is a rough separation into ribosomal RNA (fraction 1) and transfer RNA (fraction 2), but it should be pointed out that during the 2-methoxyethanol/phosphate separation ribosomal RNA is fragmented into segments of 4–8s and the variation in base composition of fraction 1 represents the variation in base composition along the length of the RNA chains. Fraction 1 was only partly soluble in solvent system 127, but since the base compositions of fractions from normal liver RNA separated in solvent system 150 (Kirby, Hastings, O'Sullivan, 1962) have shown a similar range, those reported in Table IV are a fair representation, and demonstrate again the increased solubility of fragments high in adenine in the organic phase. The increased peak in tubes 5–10 (Fig. 1 and 2) is an indication of an increased proportion of a segment high in guanine in the ribosomal RNA segments, but as this may have come about by the increased activity of a nuclease the change in the pattern may not be significant.

Fig. 3 shows that fraction 2 (the transfer RNA) is essentially the same in the tumour tissues studied. The patterns of yeast and liver transfer RNAs are almost identical with those shown having maxima about tube 40, but that these have almost no material in tubes 0–5. The fractionation is therefore of the transfer RNAs associated with different aminoacids and as shown in Fig. 3 and Table V (fractions 2/2–2/6) a real fractionation has been achieved. The material in tubes 0–5 (Fig. 3) is completely separated from the other fractions by redistribution (Fig. 4). It has an unusual base composition (Table V, fraction 2/1) and proved, by test with the diphenylamine reagent (Burton, 1956) and determination of the thiamine content, to contain DNA (Tables VI and VII). Although there is some increase in the DNA content of RNA from tumours compared with normal liver, again it seems likely that this is due to nuclease action during the isolation (breaking down the tissue prior to addition of phenol) as this DNA is not precipitated by 2-ethoxyethanol (in contrast to normal DNA); however, the DNA from the hepatoma 2/1 fraction (Table VII) has rough equivalence of G with C and A with T.

The results of these experiments show that the transfer RNA of normal and tumour tissues are probably identical and that there is a great similarity in the fragments which make up the ribosomal RNAs, indicating there is a general overall pattern of base sequences in the RNAs of the ribosomes.

The main difference found is a progressive increase in DNA but very little change in the amounts of total RNA ; that is, there is less ribosomal RNA per unit of DNA. Since ribosomes are essential for protein biosynthesis the result may indicate there is a limited biosynthesis of proteins in the tumour tissues. This conclusion would suggest that there should be a change in *m*RNA during carcinogenesis and studies in this direction are in progress.

### SUMMARY

Nucleic acids have been isolated from normal and tumour tissues and from rat liver during feeding of carcinogenic and non-carcinogenic azo-dyes. In agreement with previous workers an increase in DNA/100 g. tissue was found during the feeding of carcinogenic azo-dyes, however in contrast to previous results very little change in the RNA/100 g. tissue was found. RNA was fractionated into ribosomal fragments (fraction 1) and transfer RNA (fraction 2) and by counter-current distribution studies it appeared that the ribosomal RNA fragments were all very similar and the transfer RNAs also appeared to be almost the same. Small differences in fraction 1 and the amount of DNA in fraction 2 are probably attributed to nuclease action.

Since the amount of RNA is decreased relatively to the amount of DNA, the types of protein synthesized may well be limited in tumour tissues, indicating less of the genome is available for gene expression.

The authors wish to thank Professor A. Haddow, F.R.S., for his interest and Mr. J. Everett for synthesizing the 4′-fluoro-4-dimethylamino-azobenzene used in these experiments.

This investigation has been supported by grants to the Chester Beatty Research Institute (Institute of Cancer Research: Royal Cancer Hospital) from the Medical Research Council and the British Empire Cancer Campaign for Research, and by the Public Health Service Research Grant No. CA-03188-08 from the National Cancer Institute, U.S. Public Health Service.

M.A.S. was a recipient of Travelling Studentship of Biochemistry of the National University of Ireland.

### REFERENCES

Burton, K.—(1956) *Biochem. J.*, **62**, 315.

Kirby, K. S.—(1956) *Biochem. J.* **64**, 405.—(1957) *Ibid.*, **66**, 495.—(1960) *Brit. J. Cancer*, **14**, 147.—(1961) *Biochim. biophys. Acta*, **47**, 18.—(1962*a*) *Ibid*, **55**, 545.—(1962*b*) *Ibid* **61**, 506.

*Idem*, Hastings, J. R. B. and O'Sullivan, M. A.—(1962) *Ibid*, **61**, 978.

Miller, J. A. and Baumann, C. A.—(1945), *Cancer Res.*, **5**, 227.

*Idem* and Miller, E. C.—(1953) *in* ' Advances in Cancer Research ', (eds. Greenstein, J. P. and Haddow, A.) Vol. 1. p. 340. New York (Academic Press).

Mills, G. T. and Smith, E. E. B.—(1951) *Science*, **114**, 690.

Price, J. M., Miller, E. C. and Miller, J. A.—(1949), *Proc. Soc. exp. Biol., N.Y.*, **71**, 575.

Reid, E.—(1962) *Cancer Res.*, **22**, 398.

Schneider, W. C. and Klug, H. L.—(1946) *ibid.*, **6**, 691.